# Morphologic predictors of pathological complete response to neoadjuvant chemoradiotherapy in locally advanced rectal cancer

**DOI:** 10.18632/oncotarget.23419

**Published:** 2017-12-19

**Authors:** Chongda Zhang, Feng Ye, Yuan Liu, Han Ouyang, Xinming Zhao, Hongmei Zhang

**Affiliations:** ^1^ Department of Diagnostic Radiology, National Cancer Center/Cancer Hospital, Chinese Academy of Medical Sciences and Peking Union Medical College, Beijing, 10021, China

**Keywords:** rectal cancer, magnetic resonance imaging, pathologically complete response, neoadjuvant chemoradiotherapy

## Abstract

**Purpose:**

To evaluate the value of morphological parameters that can be obtained conveniently by MRI for predicting pathologically complete response (pCR) in patients with rectal cancer.

**Materials and Methods:**

A cohort of 101 patients was examined using MRI before and after Neoadjuvant chemoradiotherapy (nCRT). Morphological parameters including maximum tumor area (MTA), maximum tumor length (MTL) and maximum tumor thickness (MTT), as well as cylindrical approximated tumor volume (CATV), distance to anal verge (DTA), and the reduction rates were evaluated by two experienced readers independently.

**Results:**

Post-nCRT MTA and MTL, reduction rates and pre-nCRT DTA were proved to be significantly different between pCR and non-pCR with the AUCs of 0.672-0.853. The sensitivity and specificity for assessing pCR were 61.1-89.9% and 59.0-80.7% respectively. No significant correlation between pre-nCRT size measurements and pCR was obtained.

**Conclusion:**

The convenient morphological measurements may be useful for predicting pCR with moderate sensitivity and specificity. Combining these predictors with the aim of building diagnostic model should be explored.

## INTRODUCTION

Colorectal cancer is the third most common cancer and the fourth leading cause of cancer mortality worldwide. Rectal cancer accounts for approximately one third of all colorectal cancer cases [1, 2]. In recent years, neoadjuvant chemoradiotherapy (nCRT), followed by surgical treatment, has been widely used for treating patients with locally advanced rectal cancer (LARC). Prospective regression of tumor lesions caused by nCRT is beneficial for local control, surgical resection and survival rates [3–5]. Furthermore, nCRT leads to an effective tumor regression in 15% to 34% patients with pathologically complete response (pCR) based on present literature [6–10], which in turn is associated with improved local control, reduced incidence of distant metastases and a survival prognosis [6, 11, 12]. Limited resection or non-operative ‘wait and see’ policy was introduced as an alternative modality to conventionally total mesorectal excision (TME) for patients with clinical *complete* response (cCR) after nCRT [13, 14]. In a meta-analysis study, Li *et al* have compared the oncological outcomes in 251 patients with rectal cancer achieving cCR after nCRT with nonsurgical management and in 344 patients with cCR treated with radical surgery. Briefly, no differences were found in distant metastasis rates, disease-free and overall survival between the two groups [14]. Accordingly, new predictors of pCR after nCRT need to be investigated in order to avoid over treatment and to improve the quality of life.

Currently, magnetic resonance imaging (MRI) is considered the best non-invasive imaging approach for predicting pCR in patients with rectal cancer treated with nCRT [15–18]. Quantitative measurements of signal intensity obtained using T2-weighted or diffusion weighted MR images (DWI) before and after nCRT, showed to be useful for evaluation of pCR [8, 16–18]. Nevertheless, the primary disadvantage of this technique is the poor repeatability and the relatively controversial results [9, 19]. Significant correlations between reduction rate of tumor volume and pCR have been reported by some studies [19–22]. However, its calculation requires a lot of effort and is time-consuming. Therefore, the alternative and less tedious methods are more than necessary in busy clinical practice.

In this study, we evaluated the value of morphological parameters that can be obtained more conveniently by MRI for predicting pCR in patients with rectal cancers.

## RESULTS

### Surgical treatment and pathological findings

The basic clinical information of patients are displayed in Table 1. Among 101 patients included in the study, the performed surgical treatments included laparoscopic abdominoperineal resection (n=53), low anterior resection (n=42), Hartmann's procedure (n=2), extralevator abdominoperineal excision (n=1), and extended radical resection (n=3). pCR were observed in 18 out of 101 (17.8%) patients with locally advanced rectal tumor after nCRT, while other 83 patients (82.2%) received non-pCR.

**Table 1:** Distribution of the clinical characteristics in the pCR or non-pCR groups

variables/Groups	pCR	Non-pCR	*p*
Gender (n)			0.92^a^
Male	13	59	
Female	5	24	
Age, mean (SD)	54.3(12.9)	58.0(12.2)	0.25^b^
CEA, median (IQR)	2.5(3.0)	3.8(7.2)	0.14 ^c^

Note- CEA: carcinoembryonic antigen; SD: standard deviation; IQR: interquartile range.

^a^ : Chi-square test; ^b^: *t*-test; ^c^: Kruskal-Wallis test.

### MRI and clinical parameters

Table 2 summarizes the distribution of the parameters between the pCR and non-pCR groups for the two readers.

**Table 2:** Distribution of the MR morphologic parameters in the pCR or non-pCR groups for the two readers

Variables\Groups	Reader 1			Reader 2			Kappa/ICC
	pCR	Non-pCR	*p*	pCR	Non-pCR	*p*	
MTA, median (IQR)							
MTA_pre_ (mm^2^)	332.0(193.0)	399.0(276.0)	0.14^d^	394.0(181.0)	418.0(293.0)	0.72 ^d^	0.950^Δ^
MTA_post_ (mm^2^)	100.5(94.5)	198.0(153.0)	<0.001 ^d^	106.0(104.0)	213.0(178.0)	<0.001 ^d^	0.950^Δ^
MTARR (%)	75.4(28.8)	53.4(31.1)	0.02 ^d^	76.8(14.2)	50.7(21.3)	<0.001 ^d^	0.919^Δ^
MTL							
MTL_pre_ (mm), median (IQR)	36.0(9.0)	40.0(17.0)	0.12 ^d^	35.5(10.0)	40.0(17.0)	0.28 ^d^	0.977^Δ^
MTL_post_ (mm), mean (SD)	18.1(7.7)	24.8(8.29)	0.002 ^c^	17.5(8.0)	24.0(11.0)	0.02 ^c^	0.973^Δ^
MTLRR (%), mean (SD)	50.4(19.0)	36.2(17.0)	0.002 ^c^	51.7(19.7)	38.0(15.7)	0.002 ^c^	0.949^Δ^
MTT							
MTT_pre_ (mm), median (IQR)	13.5(4.0)	14.0(5.0)	0.94^d^	13.5(5.0)	14.0(4.0)	1.00 ^d^	0.953^Δ^
MTT_post_ (mm), median (IQR)	7.0(5.0)	8.0(4.0)	0.23^d^	6.5(6.0)	9.0(4.0)	0.15^d^	0.960^Δ^
MTTRR (%), mean (SD)	48.0(23.0)	36.0(19.0)	0.02^c^	51.1(22.7)	36.4(17.2)	0.003 ^c^	0.930^Δ^
CATV, median (IQR)							
CATV_pre_ (cm^3^)	12.5(9.3)	15.6(14.7)	0.14^d^	13.9(9.8)	16.4(19.3)	0.33 ^d^	0.984
CATV_post_ (cm^3^)	1.7(2.5)	5.1(5.2)	0.002 ^d^	1.6(2.4)	5.3(5.8)	0.003 ^d^	0.977
CATVRR (%),	86.3(24.0)	72.8(29.0)	0.01 ^d^	89.0(15.0)	73.0(26.0)	<0.001 ^d^	0.954
DTA, (mm), median (IQR)	39.5(26.0)	66.0(39.0)	0.01 ^d^	36.0(23.0)	65.0(40.0)	0.006 ^d^	0.987^Δ^
TP (n)			0.83^b^			0.83 ^b^	0.973^*^
1	8	28		1	30		
2	1	13	4	12			
3	4	22	5	22			
4	5	20	8	19			
CP_pre_ (n)			0.70 ^b^			0.59 ^b^	0.814^*^
1	1	3		0	4		
2	7	26	7	23			
3	5	26	6	26			
4	5	28	5	30			
CP_post_ (n)			0.05 ^b^			0.04^b^	0.873^*^
1	9	24		10	21		
2	8	27	7	31			
3	1	20	1	20			
4	0	12	0	11			

Note – MTA: maximum tumor area; MTARR: maximum area reduction rate; MTL: maximum tumor length;

MTLRR: maximum tumor length reduction rate; MTT: maximum tumor thickness;

MTTRR: maximum tumor thickness reduction rate; CATV: cylindrical approximated tumor volume;

CATVRR: cylindrical approximated tumor volume reduction rate; DTA: distance from tumor to anal verge;

TP: tumor position; CP: circumferential percentage; SD: standard deviation; IQR: interquartile range.

^a^ : Chi-square test; ^b^: Fisher exact test; ^c^: *t*-test; ^d^: Kruskal-Wallis test; ^Δ^ : inter-observer correlation coefficient (ICC);

^*^: Kappa statistics.

The median values of MTA_pre_ were 332.0±193.0 mm^2^ and 399.0±276.0 mm^2^ in the pCR and non-pCR groups for reader 1, and 394.0±181.0 mm^2^ and 418.0±293.0 mm^2^ for reader 2, respectively. There was no significant difference in the parameter of MTA_pre_ between the two groups for both readers (p=0.14, 0.72, respectively). Additionally, after nCRT, the parameters of MTA_post_ and MTARR revealed significant differences between the pCR and non-pCR groups for both readers (*p*<0.05). The inter-observer agreement was excellent with ICC values of 0.950 and 0.919, respectively.

The values of maximum tumor length and maximum tumor thickness were decreased obviously after nCRT compared to those before treatment. There were significant differences in MTL_post_ and MTLRR between pCR and non-pCR groups for the two readers (p<0.05). Moreover, significant differences were observed for the predictor of MTTRR between the two groups for both readers (p=0.02, 0.003). Nevertheless, there were no significant differences in terms of MTL_pre_, MTT_pre_and MTT_post_ for the two readers (p>0.05).

Similar to the parameters of MTA and MTL, significant differences for the predictors of CATV_post_ and CATVRR between the two groups (p=0.002, 0.01 for reader 1, and 0.003, <0.001 for reader 2) were found, while no significant difference was observed for the variable of CATV_pre_ for the two readers (p=0.14, 0.33, respectively).

There were significant differences for pre-treatment DTA between the two groups for the two readers (p=0.01, 0.006, respectively) with the excellent inter-observer agreements (ICC=0.987). The parameter of CP_post_ differs between pCR and non-pCR for reader 2 (p=0.04).

No significant differences were observed for the parameters of TP, gender, age, and pre-treatment serum CEA level between the two groups for the independent readers.

### Diagnostic performances of the significant predictors

**Figure 1:**
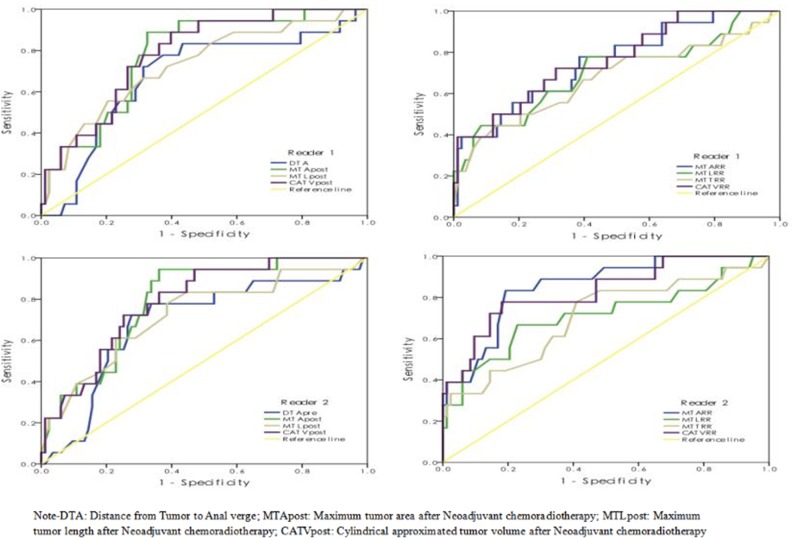
The ROC curves of selected significant parameters in predicting pCR vs. Non-pCR

The ROC curves of those significant predictors for differentiating pCR from non-pCR are shown in Figure 1, while corresponding sensitivity and specificity data are shown in Table 3. The AUCs of 0.672-0.853 were obtained for the predictors with the highest value for MTARR for reader 2 and the lowest value for MTTRR for reader 1. When the cut-off values were chosen based on the Youden index, the relative sensitivity of 61.1%-88.9% and specificity of 59.0%-75.9% were achieved, while the cut-off values were selected according to the specificity above 90.0%, the corresponding sensitivity was relatively low with the values of under 50.0%.

**Table 3:** Sensitivity and specificity of the significant parameters for predicting a pCR

	AUC	Cutoff 1	Sensitivity (%)	Specificity (%)	Cutoff 2	Sensitivity (%)	Specificity (%)
**Reader 1**							
MTA_post_ (mm^2^)	0.777	122.5	72.2	72.3	54.5	33.3	94.0
MTL_post_ (mm)	0.728	22.5	72.2	61.4	14.5	33.3	91.6
CATV_post_ (cm^3^)	0.783	2.5	72.2	73.5	0.9	33.3	94.0
DTA_pre_ (mm)	0.676	50.5	72.2	68.7	29.5	16.7	90.2
MTARR (%)	0.752	67.9	61.1	75.9	85.0	38.9	97.6
MTLRR(%)	0.701	47.5	61.1	71.1	58.5	44.4	91.6
MTTRR (%)	0.672	42.5	61.1	62.7	61.0	38.9	90.4
CATVRR (%)	0.764	79.3	72.2	68.7	93.3	38.9	98.8
**Reader 2**							
MTA_post_ (mm^2^)	0.789	134.5	72.2	69.9	52.5	33.3	94.0
MTL_post_ (mm)	0.723	21.5	77.8	61.4	14.5	38.9	90.2
CATV_post_ (cm^3^)	0.787	2.6	72.2	74.7	1.0	33.3	92.8
DTA_pre_ (mm)	0.686	48.5	72.2	72.3	29.0	11.1	90.2
MTARR (%)	0.853	65.5	88.9	69.9	85.5	38.9	98.8
MTLRR(%)	0.712	50.5	66.7	77.1	58.5	44.4	94.0
MTTRR (%)	0.702	39.0	77.8	59.0	61.5	33.3	92.7
CATVRR (%)	0.829	83.8	77.8	80.7	89.9	44.4	94.0

Note – Cutoff 1: the value with Youden index; Cutoff 2: the value with specificity above 90.0%;

MTA: maximum tumor area; MTL: maximum tumor length; CATV: cylindrical approximated tumor volume;

DTA: distance from tumor to anal verge; MTARR: maximum tumor area reduction rate;

MTLRR: maximum tumor length reduction rate; MTTRR: maximum tumor thickness reduction rate;

CATVRR: cylindrical approximated tumor volume reduction rate.

## DISCUSSION

The benefits of pCR to nCRT in rectal cancer are well elucidated. The reliable and practical predictors are thereby preventing clinicians from potentially offering less invasive treatment options. Several previous studies have indicated that the whole tumor volume after nCRT, which is calculated by multiplying each cross-section area by section thickness, and the reduction rate of whole tumor volume based on the pre- and post-nCRT MRI, are good criteria for predicting pCR [1, 17, 19, 21, 23]. However, this method is extremely time-consuming and is not suitable for routine clinical practice.

In the present study, we used the relatively convenient measurements to achieve the similar positive results. The results showed the predictors of MTA_post_, MTL_post_ and CATV_post_that was defined as multiplying MTA_post_ by MTL_post_, were useful for assessing pCR with the AUCs of 0.723-0.789, thus suggesting that the size of the residual tissue after nCRT is associated with the tumor response. The smaller post-nCRT tumor area, length and cylindrical approximated volume predicted an increased pCR rate. However no correlation was observed between tumor thickness after nCRT and pCR. A possible explanation was that, the post-nCRT tumor thickness was relatively small compared to the tumor area and tumor length. Our study found that the median value of post-nCRT tumor thickness was less than 10 mm in either pCR or non-pCR group according to both readers, hence the inevitable minor deviation that may affect the statistical result obviously. Currently, there are very few studies that have described parameters of post-nCRT tumor area or diameter for assessing a pCR. For example, Park *et al* [24] have shown that the parameter of post-nCRT tumor diameter was associated with pCR. The tumor with largest diameter that is smaller than 3 cm may predict a potential pCR (p<0.001).

Significant correlation between the reduction rates of maximum tumor area, length, thickness and cylindrical approximated tumor volume and pCR was found in the study. Among these predictors, the reduction rates of tumor area and cylindrical approximated tumor volume were relatively better compared to tumor length and thickness for identification of pCR with the AUCs of 0.752-0.853 vs. 0.672-0.712.

So for, only a few articles have reported on the relationship between tumor response and size measurement based on MR imaging, other than volumetry analysis. Kim *et al* [25] have reported that the largest diameter reduction rate of the tumor was significantly associated with pCR or near pCR after nCRT. By using the diameter reduction rate together with visual assessment, the authors obtained AUCs of 0.735-0.791 in ROC curve analysis. Additionally, another recent study evaluated the relationship between MR measurements and pathological tumor regression. Their result suggested that both the diameter and the area reduction rate are correlated with tumor regression, and the AUCs for the former predictors (0.708-0.770) were larger compared to latter ones [26]. By contrast, Patel *et al* have demonstrated that there is no consistent relation between maximum tumor length and tumor response [27].

In our study, the diagnostic performances of those significant predictors were evaluated on ROC curve analysis. The sensitivity of 61.1-88.9% and specificity of 61.4-80.7% were obtained based on the Youden index associated cutoff values for assessing a pCR. According to the previous studies, similar or slightly better results were reported by using the predictor of whole tumor volume for prediction of tumor response [14, 28, 29]. Although many authors suggested the wait-and-see strategy for the patients with clinical complete response after nCRT, the higher risk of local recurrence was reported for the patients achieving a cCR with non-surgical management compared to those achieving a pCR through radical surgery [14, 28, 29]. So, it is crucial to apply strict criteria for selecting the patients with cCR as the candidates for non-operative managements in clinical practice. Consequently, in the present study, we recommended another cutoff value with high specificity of above 90% for each predictor to select the appropriate patients to perform the nonsurgical management. It was noted that the corresponding sensitivity was relatively low. Thus specific situation of a patient like age, health status and private aspiration, should be more considered in clinical management.

The pre-treatment parameters referring to tumor size measured detected by MR imaging were not associated with higher possibility of pCR in our study, which was consistent with the previous studies based on tumor volumetry method [19, 30]. In the study published by Park *et al* [24], the pre-nCRT tumor volume was reported to be useful for evaluation of tumor response, but with limited diagnostic efficiency.

We demonstrated that the tumor distance from anal verge was proved to be significantly associated with pCR. The smaller distance to anal verge predicted a greater likelihood of pCR. Some previous studies also showed that the distance from anal verge smaller than 5 or 6 cm was correlated with favorable response [31–34]. The reason for this finding remains unclear, nevertheless one potential explanation is that the tumor close to anal verge may be related to a relative lack of organ mobility in comparison with mid and high tumors. Hence, low rectal tumors may have a reduced incidence of a geographic miss, in terms of radiation therapy volumes and a greater possibility of receiving the prescribed dose compared to the rectal tumors with higher location [31]. Nevertheless, there were several studies with contrary findings [35–37]. They showed no association found between tumor height and pathologic response [35, 36]. Patel *et al* [37] have reported that the patients with low tumors (<4 cm) were less likely to have a pCR. Still further investigation is needed to determine the relationship between distance from anal verge and tumor response to nCRT.

Several previous studies have found that lower pre-treatment serum CEA level may be a predictive factor of pCR [24, 34, 38]. Our data indicated that 83.3% of patients with pCR had a normal pre-treatment serum CEA level compared with 57.8% in the non-pCR group, but without significant difference. Additionally, Garland *et al* [36] have indicated that pre-treament serum CEA was not a reliable predictor for assessing pCR.

Parameters of tumor position, tumor circumferential percentage, patient's age and gender were not significantly correlated with pCR in the study, which was consistent with previously published data [24, 34, 39].

There were some limitations to this study. The first was not as many patients with pCR compared to those with non-pCR in a single institution study, therefore, the diagnostic performance of those predictors should be further explored in multi-central research. Second, tumor signal intensity on T2 weighted MR imaging and apparent diffusion coefficient (ADC) values on diffusion weighted imaging were not evaluated. The drawback of T2 signal and ADC measurements had poor repeatability between different equipment and different MR protocols, while the value for prediction of complete response remains controversial. The relationship between pre-CRT staging and PCR was not evaluated in this study. It was reported that a higher complete pathological response rate was observed in early T3 disease in comparison with more extensive T3 invasion [40]. Here we focused on the diagnostic performance of these morphological predictors in this study, while interesting and worth exploring, further studies on T2 signal, ADC changes and pre-CRT staging will be pursued to further improve diagnostic efficacy. Third, the tumors with mucinous components were also included in this study, and three of these lesions were detected as pCR after surgery. The reduction rate for the tumor with mucous components may be less than that without it due to the presence of mucous lake, possibly leading to misevaluation.

MR tumor regression grade (mrTRG) has been reported to be an excellent method for differentiating good from poor response in patients with rectal cancer [41–42]. Further studies need to be carried out in order to improve the interobserver consistency.

In conclusion, the convenient morphologic measurements are significantly correlated with pCR in patients with rectal cancer. The post-treatment tumor area, length, and CATV, and the corresponding reduction rate before and after nCRT are useful for predicting a pCR with moderate sensitivity and specificity. Since strict criteria are necessary for selection of candidates to participate in watch-and see strategy after nCRT, use and combination of the predictors that are needed to build the diagnostic models should be furthermore explored.

## MATERIALS AND METHODS

### Patient population

The study was approved by our institutional ethic committee. The written informed consent was obtained from each patient. A prospective study was conducted between October 2012 and August 2014. Inclusion criteria for enrollment were as follows: (1) patients diagnosed with rectal carcinoma by proctoscopic biopsy, (2) no evidence of distant metastases. CT scans of chest and abdomen were performed in all patients to exclude the possibility of metastasis. Hepatic, brain MR examination or bone scan was also performed to exclude suspicious metastases in some patients.(3) no prior treatment was applied before baseline MRI (4) no contraindications to MR examination. A total of consecutive 158 patients underwent pre-treatment MR examination. 57 patients were excluded because of the following reasons: underwent direct surgery without nCRT (n=35), incompletion of CRT (n=2), lack of MR examination during 4-6 weeks after nCRT (n=14) and the time interval between the end of nCRT and surgery beyond 6-8 weeks (n=6). Ultimately, a total cohort of 101 patients were enrolled in the study (72 men, mean age 56.3 years, range 29 to 80; 29 women, mean age 59.8 years, range 31 to 79).

### Preoperative nCRT

All patients underwent nCRT before surgery. Radiotherapy was performed by delivering 50Gy (25 fractions of 2 Gy for five times) within 35 days. Concomitant oral capecitabine was given at a dose of 1650mg/m^2^ daily for 7 periods.

### MR imaging acquisition

MR imaging was acquired on a 3.0T system (GE Discovery MR 750, General Electric Medical Systems, USA) with an eight-element phased-array wrap-around surface coil. Patients underwent rectal cleansing using Glycerin enema with a dosage of 10 ml To reduce intestinal peristalsis or rectal spasm, 10 mg of raceanisodamine hydrochloride was injected intramuscularly 20-30 min before MR examination except in patients with contraindications. The ultrasound transmission gel was administered using a rectal tube with the volume of 100-150 ml to highlight the tumor borders within the lumen. All pre- and post-CRT rectal MRI examinations were performed in the supine position by the same system. The oblique axial and sagittal T2-weighted fast-spin echo images without saturation, which were obtained orthogonal or parallel to the long axis of tumor, were regular sequences for patients in our study. Coronal T2-weighted imaging was performed to evaluate the relationship between the tumor and the levator ani muscle or anal sphincters for the patients with distal rectal cancers. The pelvic axial T1-weighted imaging, T2-weighted sequence with fat saturation and diffusion-weighted imaging were also obtained to detect the overall situation of tumor and peripheral structures. The detailed protocols are listed in Table 4. The examination protocols were stable during the study period.

**Table 4:** Protocols for the MR imaging sequences

Parameter	Oblique T2WI	Sagittal T2WI	Coronal T2WI	T1WI	T2WI/FS
TR	4800	4800	4800	5600	5700
TE	115	115	115	min	85
FOV	16	24	24	34	34
Matrix	256×320	256×320	256×320	288×224	288×224
Band-width	41	41	41	41	31
NEX	4	4	4	2	2
Frequency direction	R/L	A/P	S/I	R/L	R/L
ETL	21	21	21	4	21
Slice thickness (mm)	3	4	4	5	5
Intersection gap	0	0.4	0.4	0.5	0.5

### Imaging analysis

All data were transferred to a PACS workstation. Imaging analysis was performed independently bytwo specialist radiologists with 17 and 14 years’ experience in gastrointestinal imaging. The two readers were both blinded to the pathological and clinical information. The high-resolution T2-weighted imaging was used as the key sequence for evaluation. In case the tumor could not be discerned clearly based only on T2WI, the other sequences were used for assistance. The values were recorded by the average of two repetitive measurements by each reader. These evaluated parameters were defined specifically as follows.

#### Maximum tumor area (MTA)

**Figure 2:**
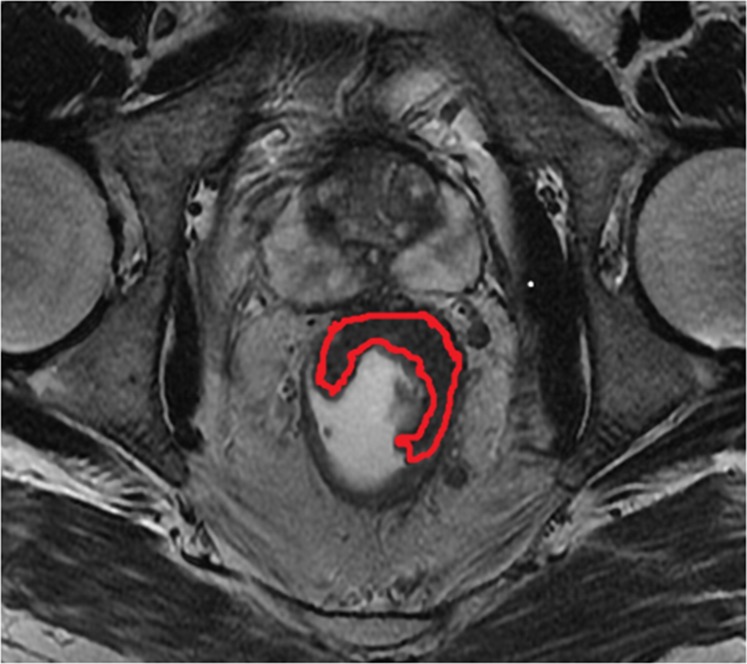
The region delineated by red curved line indicates indicates the measurement of maximum tumor area(MTA) on the oblique axial T2-weighted imaging

Maximum tumor area was measured on the section with largest tumor dimension on the oblique axial T2-weighted imaging (Figure 2). The region of interest (ROI) was manually traced along the bounder of tumor while the corresponding area was calculated automatically. The image was traced twice and the mean value was recorded by the two readers independently. The MTA was measured on MRI before and after nCRT (MTA_pre_, MTA_post_). The Maximum Tumor Area Reduction Rate (MTARR) was calculated using the following formula: (MTA_pre_-MTA_post_/MTA_pre_) × 100%.

#### Maximum tumor length (MTL) and maximum tumor thickness (MTT)

**Figure 3:**
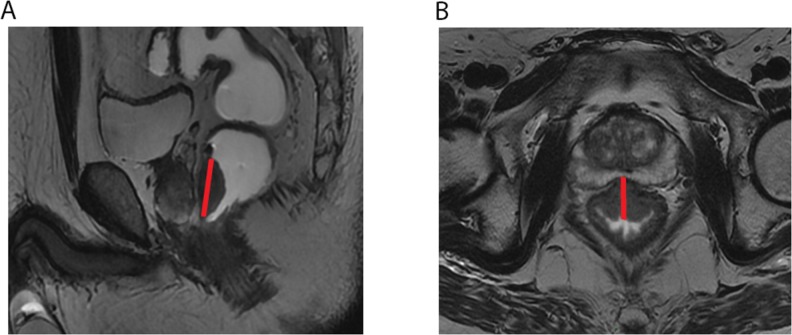
The red straight lines across the lesion indicate the measurement of maximum tumor length (MTL) **(A)** and maximum tumor thickness (MTT) **(B)** on sagittal and oblique axial T2-weighted imaging.

MTL and MTT were obtained on the section with largest tumor on sagittal and oblique axial T2-weighted imaging respectively (Figure 3A and 3B). A correspondingly bending line was used for evaluating MTL for the bending tumor. The obscure composition area at the connective band between tumor and rectal wall or other peripheral tissues was avoided during the measurements. MTL and MTT were obtained on MRI before and after nCRT and labeled as MTL_pre_, MTL_post_, MTT_pre_, and MTT_post_, respectively. The Maximum TumorLength Reduction Rate and Maximum Tumor Thickness Reduction Rate (MTLRR, MTTRR) was calculated using the following formula: (MTL_pre_-MTL_post_/MTL_pre_) × 100% and (MTT_pre_-MTT_post_/MTT_pre_) × 100% respectively.

#### Cylindrical approximated tumor volume (CATV)

A crude measurement, cylindrical approximated tumor volume (CATV) was defined as multiplying MTA by MTL on MRI before and after nCRT and recorded as CATV_pre_ and CATV_post_. The cylindrical approximated tumor volume reduction rate (CATVRR) was calculated using the equation CATVRR (%) = (CATV_pre_-CATV_post_/CATV_pre_) × 100%.

#### Distance from tumor to anal verge (DTA)

**Figure 4:**
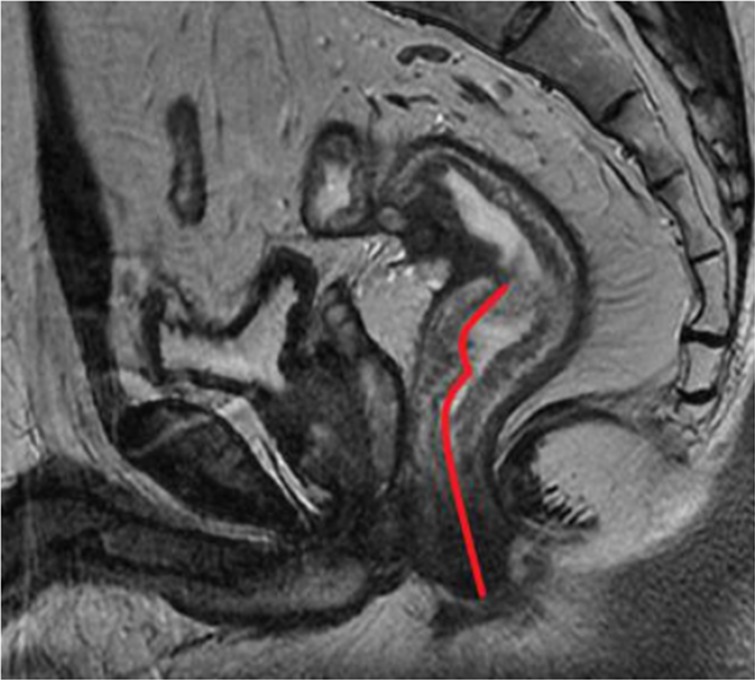
The red curved line from from the inferior part of the tumor to the anal verge indicates the measurement of distance from tumor to anal verge (DTA) on sagittal T2-weighted imaging

The DTA was measured on the pre-treatment sagittal T2-weighted imaging from the inferior part of the tumor to the anal verge (Figure 4).

#### Tumor position (TP)

**Figure 5:**
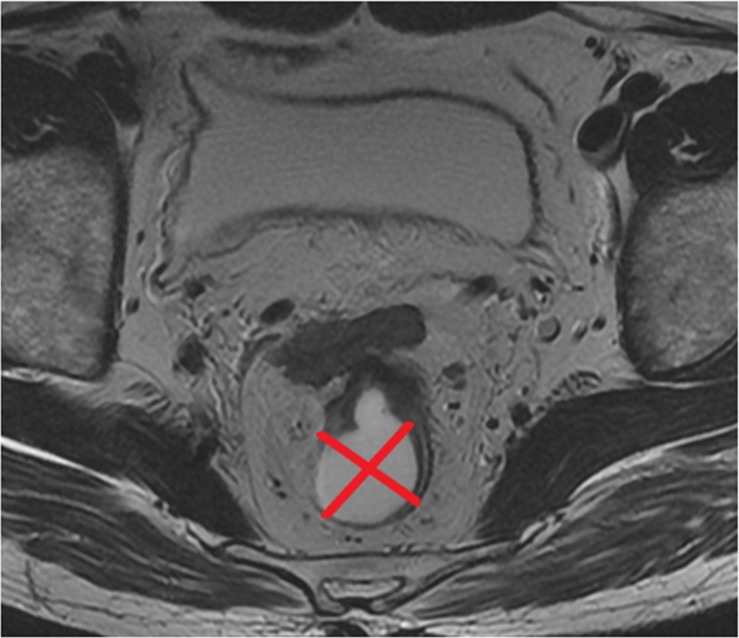
Two orthogonal lines that passed through the perceived centre of the lumen divided the rectal wall into four groups of tumor position (TP)

The Tumor position (TP) was evaluated on the same section that was used for measurement of MTA on oblique axial T2-weighted imaging. The rectal wall was divided into four parts along two orthogonal lines that passed through the perceived center of the lumen, i.e. anterior, posterior, left lateral, and right lateral wall (Figure 5). Consequently, the parameter of TP was classified into four types based on the location of the main body of the tumor (type 1, anterior wall; type 2, posterior wall; type 3, left lateral wall, and type 4, right lateral wall).

#### Circumferential percentage (CP) of rectal involvement

Circumferential Percentage (CP) of rectal involvement was categorized as four groups according to the percentage of the circumference of rectum involved by the tumor (group 1, <25%; group 2, 25-50%; group 3, 50%-75%; and group 4, >75%). The evaluations were performed on the oblique axial T2-weighted images before and after nCRT and recorded as CP_pre_and CP_post_ by the independent readers.

Besides the imaging parameters, some clinical data including gender, age and pre-treatment carcinogen-embryonic antigen (CEA) level were also recorded for evaluation.

### Pathological assessment

For all the patients the surgery was performed 6-8 weeks after nCRT. The specimen was fixed in formalin for 24 hours, then each specimen was sliced transversely, perpendicular to the rectal lumen, at 3 mm intervals. The tumor and lymph nodes slices were stained using hematoxylin-eosin. Each specimen was analyzed precisely by a single experienced pathologist with 19 years of experience in colorectal histopathology. pCR was defined as no residual tumor cells found on histological examination of the specimen (T0N0).

### Statistical analysis

All of the qualitative data were compared with Chi-square test between pCR and non-pCR groups for each reader. Kolmogorov-Smirnov test was used for normal distribution test for the quantitative data. The normally distributed data were expressed as mean±standard deviation, SD and nonparametric data as median±interquartile range, IQR. The independent sample *t*-test was used for parametric continuous variables to determine the significant difference between pCR and non-pCR groups for each reader. The chi-square test or Fisher exact test was used for the nonparametric continuous variables. P<0.05 was considered to be statistically significant.

The diagnostic performance was done using receiver operating characteristic (ROC) curves for those significant predictors, and areas under the receiver operating characteristic curve (AUCs) were calculated. For each predictor, a cut-off value associated with Youden Index was obtained, and the sensitivity and specificity were calculated. Furthermore, another cut-off value with specificity above 90.0% was also evaluated in consideration of the policy that strict criteria should be applied in selecting patients for the “wait-and-see” strategy.

Inter-observer agreement between two readers was assessed by using Kappa statistics for qualitative data, and inter-observer correlation coefficient (ICC) for quantitative data. The relative criteria were as follows: < 0.20, low agreement; 0.21-0.40, fair; 0.41-0.60, moderate; 0.61-0.80, substantial; and greater than 0.80, excellent agreement.

Statistical Package for the Social Sciences (SPSS, version 20.0, Inc., Chicago, IL) was used to perform the statistical analyses.

## Abbreviations

pCR: Pathologically complete response
non-pCR: Pathologically incomplete response
nCR: Neoadjuvant chemoradiotherapy
MTA: Maximum tumor area
MTL: Maximum tumor length
MTT: Maximum tumor thickness
CATV: Cylindrical approximated tumor volume
DTA: Distance to anal verge
LARC: Locally advanced rectal cancer
TME: Total mesorectal excision
cCR: Clinical *complete* response
MRI: Magnetic resonance imaging
DWI: Diffusion weighted MR images
MTARR: Maximum Tumor Area Reduction Rate
MTLRR: MaximumTumorLength Reduction Rate
MTTRR: Maximum TumorThickness Reduction Rate
CATVRR: Cylindrical approximated tumor volume reduction rate
TP: Tumor position
CP: Circumferential Percentage
CEA: Carcino-embryonic antigen
ROC: Receiver operating characteristic
AUCs: Areas under the receiver operating characteristic curve
ICC: Interobserver correlation coefficient
ADC: Apparent diffusion coefficient
